# Transcription profiling in papillary thyroid carcinoma reveals potential diagnostic markers and drug targets

**DOI:** 10.1186/1753-6561-7-S2-P51

**Published:** 2013-04-04

**Authors:** Mateus CB Filho, Fabio A Marchi, Silvia R Rogatto, Luiz P Kowalski

**Affiliations:** 1 International Research Center/AC Camargo Hospital, Sao Paulo, Brazil; 2 Sao Paulo University, Sao Paulo, Brazil; 3 Faculty of Medicine/UNESP, Botucatu, SP, Brazil

## Background

Papillary thyroid carcinoma (PTC) is the most frequent malignant endocrine neoplasia with an increasing prevalence in the last decades. We aim to identify transcripts and pathways associated with PTC tumorigenesis.

## Materials and methods

RNA from tumor and adjacent normal samples was evaluated using Sure Print G3 8x60K slides (Agilent Technologies). Sixty-five tumor (T) and four normal (N) tissues were labeled with Cy5. A pool composed by nine normal samples (without the corresponding tumor assayed) was labeled with Cy3 and used in the co-hybridization. Statistical analysis was performed using two approaches, a paired (4N vs 4T) and an independent analysis (9N vs. 61T).

## Results

Overlapping paired (paired Significance Analysis of Microarray with 3% False Discovery Ratio) and independent analysis (mean log ratios <-1 or >1 with 99% Confidence Interval) resulted in a list of 546 deregulated genes. Networks and functional analysis were generated through IPA software (Ingenuity^®^ Systems). The major molecular network identified was related to endocrine system development and function and down regulation of tyrosine metabolism was the main canonical pathway. A preliminary validation was carried out with RT-qPCR for *HMGA2.* A higher expression was confirmed (*P*<*0.001*) in an independent sample set (11N vs. 47T). *HMGA2* expression had also diagnostic ability, correctly classifying 117/121 samples according to tumor status (sensibility=97%, specificity=94% and area under the ROC curve=0.989).

## Conclusion

This study unveils transcription modulations during PTC genesis and *HMGA2* may be a potential diagnostic marker. Functional studies are required to confirm *HMGA2* as an oncogenic driver in PTC and with a possible role as a drug target.

## Financial support

FAPESP and CAPES.

